# Is human classification by experienced untrained observers a gold standard in fixation detection?

**DOI:** 10.3758/s13428-017-0955-x

**Published:** 2017-10-19

**Authors:** Ignace T. C. Hooge, Diederick C. Niehorster, Marcus Nyström, Richard Andersson, Roy S. Hessels

**Affiliations:** 1 Experimental Psychology, Helmholtz Institute, Utrecht University, Heidelberglaan 1, 3584 CS Utrecht, The Netherlands; 2 Lund University Humanities Lab, Lund University, Helgonabacken 12, 22362 Lund, Sweden; 3 Department of Psychology, Lund University, Lund, Sweden; 4 Eye Information Group, IT University of Copenhagen, Copenhagen, Denmark; 5 Department of Philosophy and Cognitive Sciences, Lund University, Lund, Sweden; 6 Developmental Psychology, Utrecht University, Helgonabacken 1, 3584 CS Utrecht, The Netherlands

**Keywords:** Fixation classification, Eye tracking, Human coder

## Abstract

Manual classification is still a common method to evaluate event detection algorithms. The procedure is often as follows: Two or three human coders and the algorithm classify a significant quantity of data. In the gold standard approach, deviations from the human classifications are considered to be due to mistakes of the algorithm. However, little is known about human classification in eye tracking. To what extent do the classifications from a larger group of human coders agree? Twelve experienced but untrained human coders classified fixations in 6 min of adult and infant eye-tracking data. When using the sample-based Cohen’s kappa, the classifications of the humans agreed near perfectly. However, we found substantial differences between the classifications when we examined fixation duration and number of fixations. We hypothesized that the human coders applied different (implicit) thresholds and selection rules. Indeed, when spatially close fixations were merged, most of the classification differences disappeared. On the basis of the nature of these intercoder differences, we concluded that fixation classification by experienced untrained human coders is not a gold standard. To bridge the gap between agreement measures (e.g., Cohen’s kappa) and eye movement parameters (fixation duration, number of fixations), we suggest the use of the event-based F1 score and two new measures: the relative timing offset (RTO) and the relative timing deviation (RTD).

Most raw data from observation tools (MRI scanners, video cameras, microphones, motion trackers, eye trackers) only become useful if parts of the data are classified into meaningful units. One may think of signs in sign language extracted from video (Kita, van Gijn, & van der Hulst, 1998), automatic word recognition from a voice recording (Cooke, Green, Josifovski, & Vizinho, 2001) and saccade and fixation categorization in eye-tracker data (Nyström & Holmqvist, 2010). In research fields in which large amounts of data have to be processed much effort is put in automated classification. Recently we published an article about a noise-robust fixation detection algorithm designed to deal with eye-tracking data from infants (Hessels, Niehorster, Kemner, & Hooge, 2016b). During the review process one of the reviewers asked us to evaluate our algorithm against “the golden standard of manual classification.” To many this may seem a reasonable request, but at the time we did not see the added value of manual classification because we developed our noise-robust fixation detection algorithm specifically to avoid human coding. Our reasoning was that manual classification of eye-tracking data is expensive, slow (as compared to a computer) and prone to subjective biases and should therefore be avoided. In other words, we did not acknowledge human classification as a gold standard for fixation detection in the eye-tracking signal. To be clear what we mean by gold standard,^1^ we use the definition of Versi (1992): “The gold standard is not the perfect test but merely the best available test” (p. 187). This means that after a new technical development one gold standard may be replaced by another one. The gold standard in mobile eye tracking for mapping point of regard to the world with static stimuli may soon change to a new one. Previously such analysis was done by hand, but recent developments, however, made automated 3-D mapping of gaze to the world in simple controlled static environments available (Pfeiffer, Renner, & Pfeiffer-Leßmann, 2016). In many research fields manual classification is still the gold standard. In the literature we can find many different applications of and rationales for the standing of human manual classification.

Why would one want to do manual classification of scientific data instead of using an algorithm? There are various reasons to use manual classification. Manual classification can be used in three ways:

1. ***Manual classification to process data*** Some classification tasks have to be done by hand because automated protocols simply do not exist (yet). These tasks include the classification of stimuli, behavior and responses and are used in many fields (e.g., political science, computer science, linguistics, psychology, and biology). Some examples of tasks that require(d) human classification are because an automated protocol is/was not available: video observation of human behavior (Ozonoff et al., 2010), microsaccade detection in an eye-tracking signal from an analogous eye tracker (Steinman, Cunitz, Timberlake, & Herman, 1967) or gaze mapping of eye-tracking data from a head-mounted mobile eye tracker (Foerster, Carbone, Koesling, & Schneider, 2011; Gidlöf, Wallin, Dewhurst, & Holmqvist, 2013; Hayhoe, Shrivastava, Mruczek, & Pelz, 2003; Land, Mennie, & Rusted, 1999).
2. ***Manual classification to validate algorithms*** Manual classification is a common method to test algorithms in research fields in which automated classification is possible. Andersson, Larsson, Holmqvist, Stridh, and Nyström (2017) wrote: “A Human–Algorithm comparison, however, often assumes that humans behave perfectly rationally and that, consequently, any deviation from perfect agreement is due to the mistakes of the algorithm” (p. 619). The procedure is often as follows; two or three human coders and the algorithm classify a significant quantity of data. Then algorithm classifications are compared to human classifications using measures such as Cohen’s kappa (Cohen, 1960). If there is enough agreement between the human classification and the algorithm classification, the algorithm is considered good enough. We will refer to this whole procedure as the strict gold standard approach. An example of this is found in Munn, Stefano, and Pelz (2008) who developed an algorithm to classify fixations produced during the viewing of a dynamic scene. They state the following about their algorithm: “In comparing the performance of this algorithm to results obtained by three experienced coders, the algorithm performed remarkably well.” In Zemblys, Niehorster, Kolmogortsev, and Holmqvist (2017) we found an interesting quote concerning human classification: “We did not have multiple coders to analyze interrater reliability, as this would open another research question of how the coder’s background and experience affect the events produced.” By using only one coder Zemblys et al. can still apply the strict gold standard approach. However, it is interesting that they expect coders to produce different results.
3. ***Manual classification to teach artificial intelligence and to develop algorithms*** Manual classifications may yield a lot of information that may be useful to include in algorithms. This can be done implicitly or explicitly. In a sign language study, Kita, van Gijn, and van der Hulst (1998) tested a new coding scheme containing criteria. They had two human coders analyze signs and co-speech gestures that are produced in natural discourse. Based on the good agreement between the classifications they conclude: “These criteria can be used for the technology of automatic recognition of signs and co-speech gestures in order to segment continuous production and identify the potentially meaning-bearing phase” (p. 23). This was an example of the use of explicit knowledge. Machine learning is a method to use implicit knowledge contained by the manual classifications. Tigges, Kathmann, and Engel (1997) used an artificial neural network (ANN) to detect saccades during smooth pursuit in an EOG signal. The results were tested against three human coders and they conclude: “A total of 1,354 possible saccadic events were identified and classified by three experts on the basis of a consensus rating to have a gold standard for the training and testing of the ANN” (p. 177). This is interesting because the authors also write: “There are no definite rules that could be used for a knowledge based identification algorithm for an automated analysis” (p. 176). They use an opaque detector (the ANN), which does not allow insight into the logic of the internal algorithm (i.e., one does not really know what implicit “rules” it has developed) and test it against three human coders for whose internal algorithms are equally unknown.

## Is human classification good enough to be a gold standard?

Many applications of human classifications are done under the strict gold standard approach. However, there is debate whether this approach is valid; perhaps the current gold standard (of manual classification) may not to be the best tool available. Mikhaylov, Laver, and Benoit (2012) investigated coder reliability and misclassification in manual classification of party election programs. They wrote: “Our findings indicate that misclassification is a serious and systemic problem with the current CMP^2^ data set and coding process, suggesting the CMP scheme should be significantly simplified to address reliability issues” (p. 78). Larsson, Nyström, Andersson, and Stridh (2015) wrote: “The fact that the two experts sometimes differ makes it even harder to decide which one to trust or use as the ‘gold standard’” (p. 151). Others question whether human coders have the cognitive potential to act as a gold standard for some coding tasks. Salvucci and Anderson (2001) claimed that human coding of eye movement protocols is impossible because humans cannot interpret the data (which may consist of hundreds of protocols) consistently, accurately, and in a reasonable amount of time. The coding interface may have an influence on the classification process. The nature of these interfaces may range from the replay of raw signals to dedicated software to visualize the different aspects of the raw signals. In real-world eye-tracking studies (e.g., Foerster et al., 2011; Hayhoe et al., 2003; Land et al., 1999), the human rater often codes the events on the basis of a scene video with superimposed gaze position for which the interrater reliability might differ from other interfaces. Moreover, the coding of saccades and fixations on the basis of a video of the eye might lead to even bigger differences in interrater reliability. In our own study (Hessels, Niehorster, et al., 2016b), we wrote: “It should be noted, however, that the expert coders did not produce identical outcome measures, such that the question becomes how informative one expert coder actually is. Future research should examine whether expert coders serve as a good gold standard for event-detection algorithms” (p. xxx). Even Andersson et al. (2017), who applied the gold standard approach, wrote: “Human coders are not perfect and there are indeed difficult classification cases, but the general sentiment is that, in the simple case, what is a fixation and what is a saccade is something we can agree on” (p. 634).

## The present study

Although Andersson et al. (2017) suggest that we can agree in simple cases, we do not know if this is the case. Researchers investigating microsaccades probably have a different (implicit) definition of a saccade than do eye movement researchers investigating gaze behavior to faces. One can expect that the latter are less interested in small saccades but rather in saccades that bring gaze from the left eye to the mouth. The problem of vague or implicit definitions is not unique for eye movement research, in medical diagnosis it is known that individual coders may classify differently. Wing, Cooper, and Sartorius (1974) write in their instruction manual for classification of psychiatric symptoms: “This manual is a particular method of standardizing the elements of the diagnostic process with a view to achieving comparability between clinicians. The most important part of this book is therefore the glossary of definitions of symptoms” (p. xxx). Our question is, Do the classifications of a group of experienced but untrained human coders agree? And if so, how? Or if not, what are the differences between the classifications? Related questions are whether human coders are prone to floating criteria; do they apply similar criteria after coding for thirty min? Do researchers of the same lab who work with the same algorithms classify in the same way? From here on when we write human coders, we refer to experienced untrained human coders. We invited 13 eye movement researchers and had them manually classify about 6 min of eye-tracking data with a mouse and a simple coding interface. The eye-tracking data was collected with a Tobii TX300 (300 Hz) and derived from infants and adults. To our knowledge a systematic study with more than three human coders has not been done yet in the field of eye tracking. We compared event parameters like fixation duration, number of fixations and intercoder sample-by-sample reliability with Cohen’s kappa (Cohen, 1960). On the basis of our results, we developed a new agreement measure. We also characterized the human coders by modeling their putative criteria for fixation onset, fixation offset, and minimum saccade amplitude. One may ask why we think that a saccade amplitude criterion may play a role in a fixation classification task. Firstly, the eyes are never still when subjects are instructed to fixate, small eye movements may occur (Martinez-Conde, Macknik, & Hubel, 2004; Steinman et al., 1967). Secondly, in many definitions of a fixation, small saccades are accepted as part of the fixation, meaning that they are not classified as saccades cutting a fixation in two parts. For example, Hooge and Erkelens (1999) allowed saccades up to 2.1° to be part of a fixation. It may depend on the (implicit) criteria of the human coder whether variations in the position signal are acknowledged as saccades or as part of the fixation.

In this article we will answer the question whether classification by untrained but experienced human coders is the gold standard of fixation detection. It should further be noted that currently in the eye-movement field, human classification by experienced untrained observers is believed to be the gold standard for event classification, as evidenced in the introduction. In this article, we simply investigated whether human classification following this standard practice approach indeed is the gold standard. To do so we will use measures extracted from the raw classifications (agreement measures, eye movement measures and estimated putative thresholds). Furthermore, irrespective of the answer to the previous question, we will discuss the standing of human classification in modern eye tracking.

## Method

### Coders

We engaged 13 eye-tracking researchers in the fixation labeling task. We removed one human coder from the analysis because we found out he had never looked at raw data before. In addition, the percentage of samples he coded as fixations was 53.2%, which is 2.9 standard deviations below the group average of 69.7% (*SD* = 5.6%). The average percentage after removal was 71.1% (*SD* = 2.7%). The remaining 12 coders are members from different research groups; details about them may be found in Table 1. Written informed consent was provided by the coders, and the experiment was conducted in accordance with the Code of Ethics of the World Medical Association (Declaration of Helsinki).

**Table 1:** Details about the 12 experts who classified fixations

	Name	Age (yr)	Exp (yr)	Affiliation	Subject Group	Event	Algorithm	Eye Tracker
D.N.	Diederick Niehorster	29	10	Humlab Lund	Healthy adults, Asians	SP, fix, sacc	NH2010, SH2003, I2MC	EL1000(+), SMI RED-m, Tobii 2150
I.H.	Ignace Hooge	50	24	Exp Psy Utrecht	Healthy adults	all	HC2013	Coils, EL2, EyeTech TM3, Tobii's, Pupil Labs
R.H.	Roy Hessels	26	4	Dev Psy Utrecht	Infants, healthy adults	fix	I2MC	Tobii TX300, SMI RED60/120
J.V.	Jacco van Elst	37	10	Dev Psy Utrecht	Dyslexics, healthy adults	sacc, fix, verg	SH2003	LC, Tobii TX300
J.B.	Jeroen Benjamins	36	4	Exp Psy Utrecht	Healthy adults	fix	SH2003, HC2013	EL2, Tobii Eye trackers, Pupil Labs
P.Z.	Paul Zerr	29	3	Exp Psy Utrecht	Healthy adults	sacc	SR Research	EL1000(+)
M.S.	Martijn Schut	26	2	Exp Psy Utrecht	Healthy young adults	sacc fix	NH2010	EL1000(+), Eye Tribe
J.F.	Jasper Fabius	27	2	Exp Psy Utrecht	Healthy adults, stroke patients	sacc, fix	NH2010, SR Research	EL1000(+), Eye Tribe
K.H.	Kenneth Holmqvist	52	22	Humlab Lund	Healthy adults, clinical groups	sacc, fix, SP	many	DPI, many SMIs, EL1000
M.N.	Marcus Nyström	38	14	Humlab Lund	Healthy adults, patients	all	NH2010, EK2003	SMI Hispeed, SMI RED, EL1000(+)
R.A.	Richard Andersson	37	12	Humlab Lund	Healthy adults	all	SMI, EK2003, NH2010	SMI Hispeed, EL1000(+)
T.C.	Tim Cornelissen	28	5	SGL Frankfurt	Healthy adults	fix, sacc	NH2010, SR Research	EL1000(+), SMI HiSpeed 240, SMI REDm

NH2010 = Nyström and Holmqvist (2010); SH2003 = Smeets and Hooge (2003), I2MC = Hessels, Niehorster, et al. (2016); HC2013 = Hooge and Camps (2013); EK2003 = Engbert and Kliegl (2003). EL1000 refers to the SR Research EyeLink1000; EL2 refers to SR Research EyeLink 2. LC refers to the LC technologies EyeGaze. SGL Frankfurt refers to Scene Grammar Lab, Goethe University Frankfurt. SP refers to smooth pursuit, fix refers to fixations, sacc refers to saccades, verg refers to vergence.

### Stimulus

The eye-tracking stimulus set consists of 70 trials of eye-tracking data measured with a Tobii TX300 at 300 Hz. We used eye-tracking data measured from the left eye. Ten of the 70 trials contained 150.1 s of eye-tracking data of two adults looking at Roy Hessels’s holiday pictures taken in the arctic area around Tromsø, Norway. The other 60 trials contained 202.1 s of eye-tracking data of infants performing a search task (Hessels, Hooge, & Kemner, 2016c). Description of the stimulus data can be found in Table 2. Precision was computed with a moving-window method applied to the entire signal. We computed the RMS deviation per window (31 samples, 103.33 ms), took the median RMS deviation per trial and averaged this over all trials.

**Table 2:** Properties of the stimulus data sets

Stimulus	Adult	Infant
Number of Samples	45,018	60,623
Duration (s)	150.1	202.1
Prop. data loss	0.02	0.23
RMSx (deg)	0.16	0.21
RMSy (deg)	0.24	0.27
RMS (deg)	0.32	0.36
Min RMS (deg)	0.23	0.18
Max RMS (deg)	0.41	1.28

The RMSx of the Tobii TX300 is remarkably lower than RMSy

**Fig. 1:**
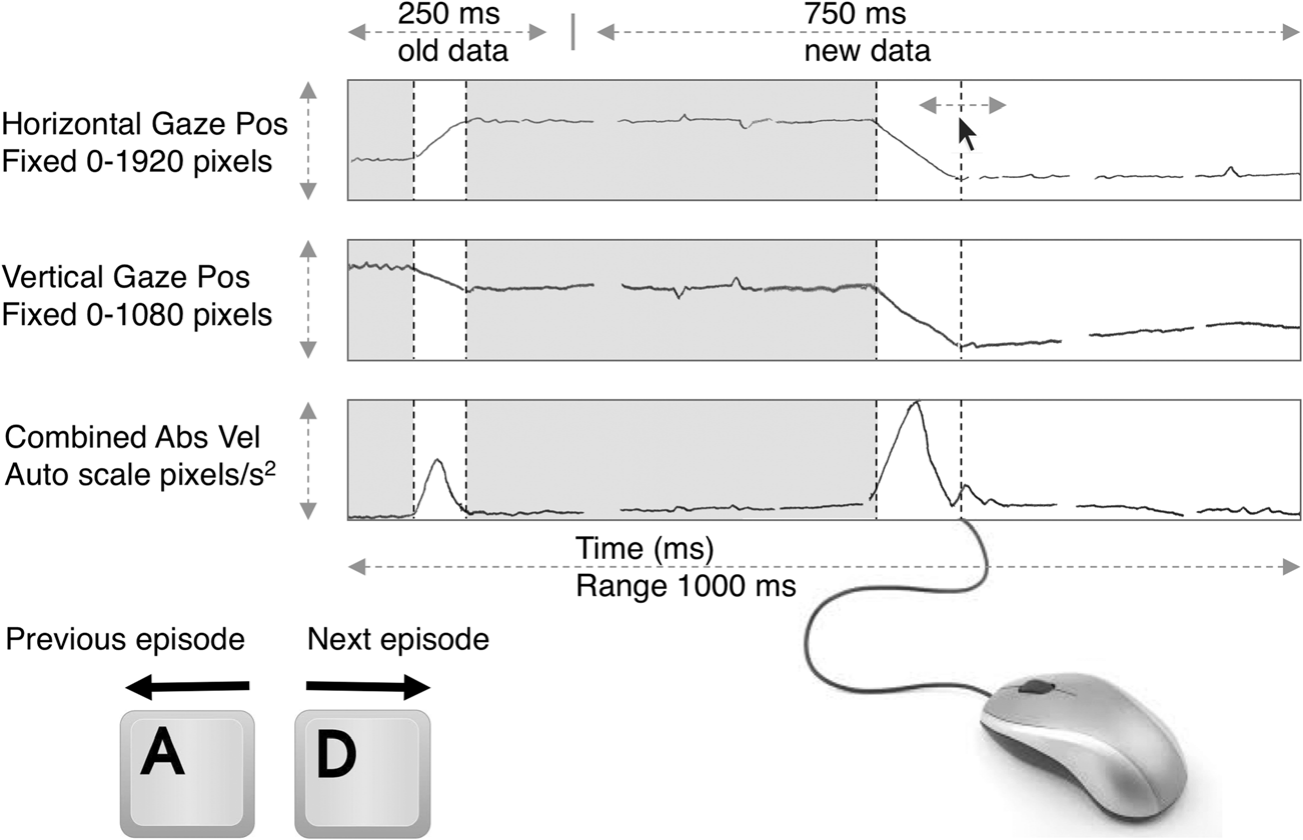
Coding interface with example data. The graphical user interface consists of three panels (horizontal gaze position, vertical gaze position, and absolute velocity). The *y*-axis of velocity is autoscaled to the largest velocity in the display, and the other two vertical axes are fixed. The display contains 1,000 ms of data (250 ms of data from the previous display, 750 ms of new data). Navigation back and forth in time is done with the “a” and “d” keys, and fixation start and end are indicated by the mouse. Already classified fixations are colored light gray in the real interface. Earlier settings can be modified and removed with the mouse

Trials of both the adult and the infant eye-tracking datasets were presented in random order on a 24-in. TFT screen (1,920 × 1,200 pixels) with a program written in MATLAB using the Psychophysics Toolbox (Brainard, 1997). The coding graphical user interface consisted of three panels (Fig. 1). The top panel showed horizontal gaze position in pixels versus time, the center panel showed vertical gaze position in pixels versus time, and the bottom panel showed velocity in pixels per second. Velocities in both the horizontal and vertical components were obtained by fitting a parabola through seven consecutive data points of the position signal (same method as in Hooge & Camps, 2013). The derivative of this parabola was used to estimate the value of the velocity of the fourth (center) data point. We computed the velocity signal by taking the vector sum of the horizontal and vertical velocity signals. The vertical axis of the position signals was fixed (respectively, 0–1,920 and 0–1,080 pixels, since measurements were done on the HD screen of the TX300). The vertical velocity axis of the velocity panel was scaled to the maximal velocity in the window. Each screen showed 1 s of data and contained the last 250 ms of the previous display (to provide context) and 750 ms new data at a time.

### Task

The experts were asked to label fixations. As context, we said that fixation locations and fixation durations were going to be used for assessing the temporal and spatial aspects of looking behavior. They could use the “a” and “d” keys to navigate back and forth through the data with steps of 1 s. With a mouse click they could indicate the start and end of a fixation and after labeling a fixation it colored light gray. The coders could delete their settings by double-clicking them. They could move their settings back and forth in time by clicking followed by dragging.

## Results

### Characteristics of the coding process

**Fig. 2:**
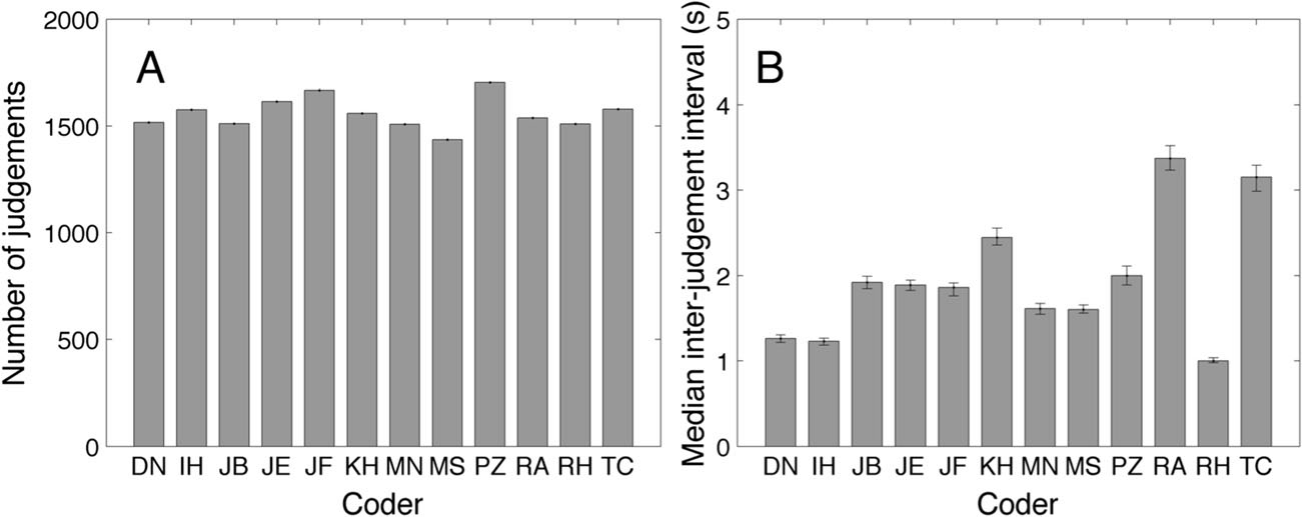
(**a**) Numbers of judgments. (**b**) Median interjudgment intervals. We report the median because the distribution of interjudgment intervals is skewed. Error bars denote the 95% confidence intervals derived from a bootstrapping procedure with 10,000 repetitions

Besides the judgments, we also logged time stamps of the classifications. Figure 2a shows the numbers of judgments during the whole session. The lowest number of judgments was 1,436 (M.S.), and the highest was 1,703 (P.Z.). The median interjudgment interval (time between two judgments) ranged from 1.00 s (R.H.) to 3.37 s (R.H.) (Fig. 2b). One might expect that more careful inspection may lead to more and more detailed events being classified. This is not the case; the number of judgments is not significantly correlated with the median interjudgment interval (*r* = .1891, *p* = .5561).

### Agreement measures

To determine whether human classification is a gold standard in fixation detection, we investigated to what degree the classifications agree. We computed Cohen’s kappa (Cohen, 1960) for all human coder pairs because many recent comparison studies use this measure (Andersson et al., 2017; Larsson, Nyström, Andersson, & Stridh, 2015; Larsson, Nyström, & Stridh, 2013; Zemblys et al., 2017). Cohen’s kappa is a sample-based measure to quantify the agreement between two coders. Cohen’s kappa takes into account and compensates for agreement based on chance. Cohen’s kappa ranges from –1 to 1. Cohen’s kappa = 0 equals chance, and Cohen’s kappa = 1 equals perfect agreement. According to Table 3, 81.9% of the values for Cohen’s kappa are higher than .8, indicating “almost perfect agreement” according to Landis and Koch (1977). The other values (18.1%) are higher than .735, and that is still substantial agreement (Landis & Koch, 1977). 9.7% of the values are higher than .9. Especially the settings of R.H., I.H., and D.N. are in good agreement with each other. The highest mean (*k* = .879) is found for coder M.N., and the lowest (*k* = .792) is found for coder P.Z.

**Table 3:** Sample-based Cohen’s kappa values for the 12 human coders

	DN	IH	JB	JF	JV	KH	MN	MS	PZ	RA	RH	TC	Mean
DN		.91	.88	.85	.88	.87	.89	.89	.77	.86	.92	.83	.868
IH	.91		.84	.81	.83	.83	.85	.84	.74	.82	.90	.81	.833
JB	.88	.84		.88	.89	.88	.90	.89	.79	.86	.89	.84	.867
JF	.85	.81	.88		.89	.87	.89	.88	.81	.85	.84	.83	.854
JV	.88	.83	.89	.89		.89	.91	.90	.81	.87	.87	.84	.873
KH	.87	.83	.88	.87	.89		.90	.88	.81	.86	.87	.86	.866
MN	.89	.85	.90	.89	.91	.90		.90	.80	.88	.90	.84	.879
MS	.89	.84	.89	.88	.90	.88	.90		.80	.87	.88	.84	.871
PZ	.77	.74	.79	.81	.81	.81	.80	.80		.78	.77	.83	.792
RA	.86	.82	.86	.85	.87	.86	.88	.87	.78		.85	.83	.849
RH	.92	.90	.89	.84	.87	.87	.90	.88	.77	.85		.83	.867
TC	.83	.81	.84	.83	.84	.86	.84	.84	.83	.83	.83		.835

The most rightward column contains the mean Cohen’s kappa for each coder.

### Eye movement parameters

**Fig. 3:**
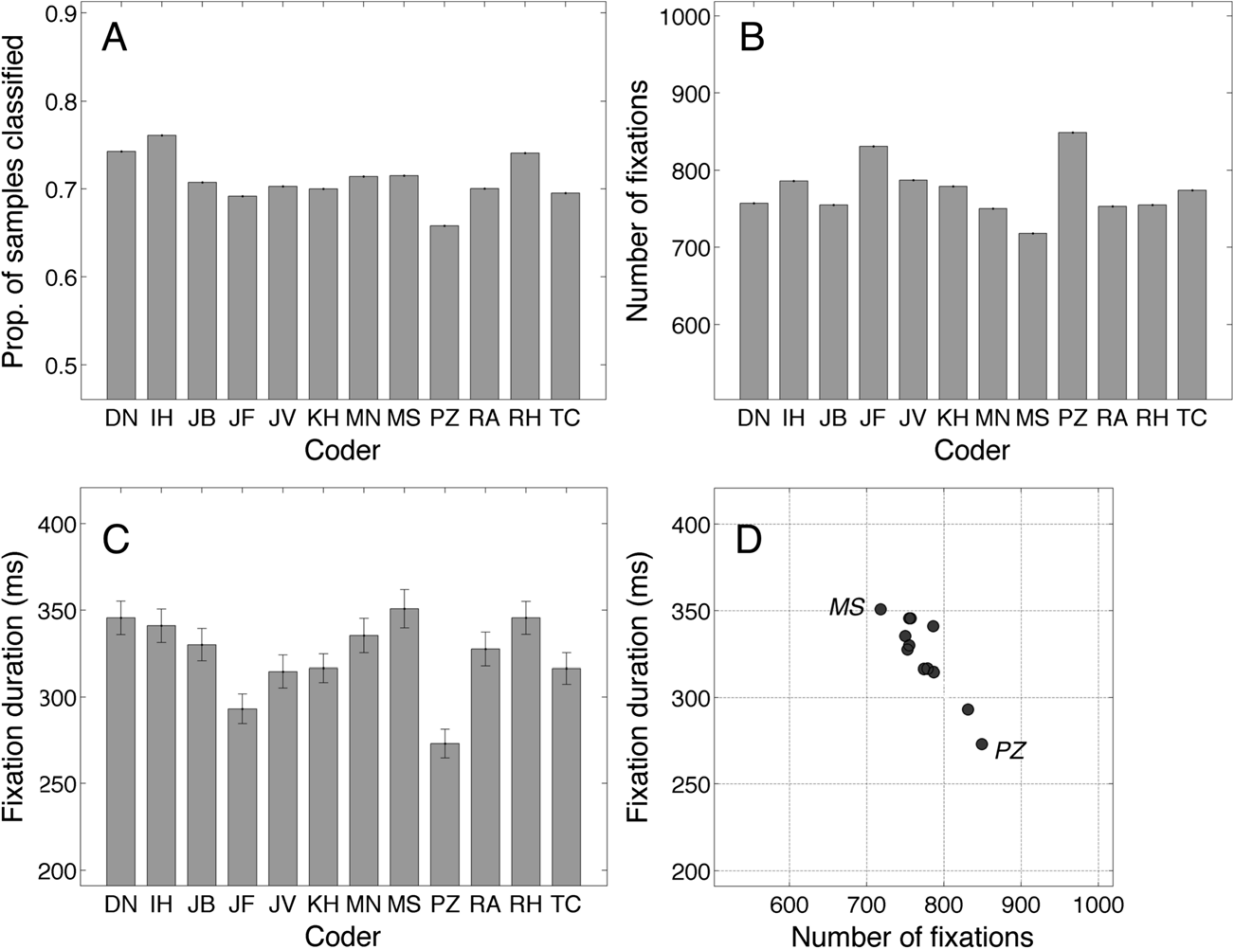
(**a**) Proportions of samples classified as fixations. (**b**) Numbers of fixations. (**c**) Fixation durations. Error bars denote standard errors of the means. (**d**) Fixation durations versus number of fixations (*r* = –.905, *p* < .001). Each point represents the data of one coder

Next we report measures that are meaningful for eye movement researchers. On the basis of the human classifications, we can calculate the proportion of samples classified as fixations, numbers of fixations, and mean fixation durations. Figure 3a contains the proportions of samples classified as fixations, ranging from .66 to .76. This number may vary between the coders, because they may have used different criteria for the start and the end of the fixation, minimal fixation duration, minimum saccade amplitude and the periods without data that are accepted as fixation. The number of fixations ranged from 718 (M.S.) to 849 (P.Z.), and the mean fixation duration ranged from 273 ms (P.Z.) to 351 ms (M.S.). Because the proportions of samples classified did not vary a lot between the human coders, it is not surprising that the coder with the highest number of fixations had the shortest fixation duration (Fig. 3d), or that the coder with the lowest number of fixations had the longest duration. Fixation duration and number of fixations have a high negative correlation (*r* = –.905, *p* < .001).

### Modeled parameters: Velocity threshold

**Fig. 4:**
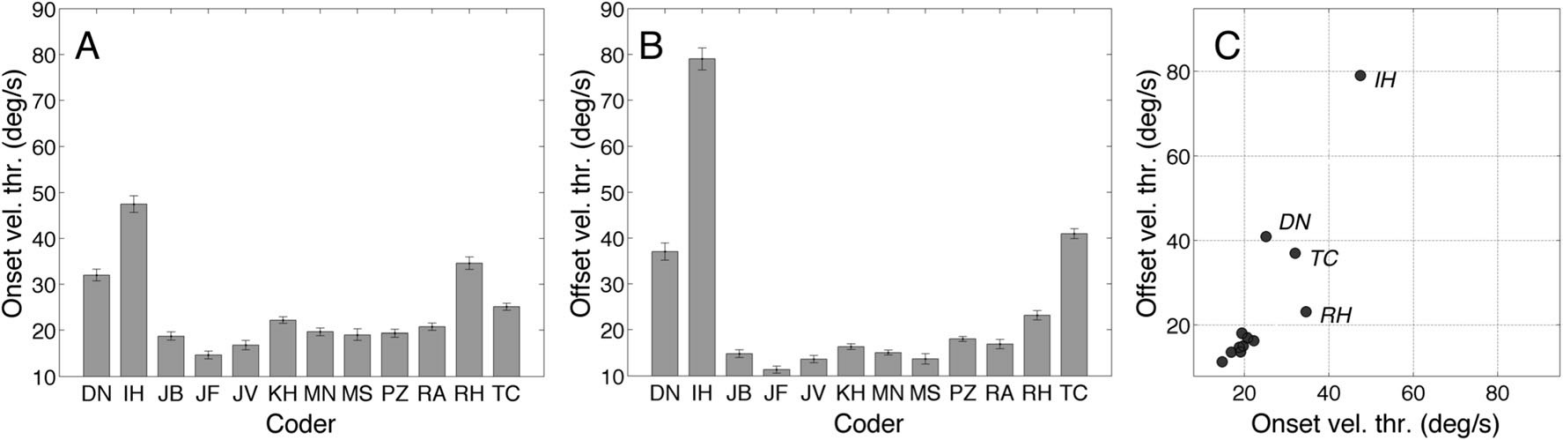
(**a**) Velocity threshold for fixation onset. (**b**) Velocity threshold for fixation offset. The error bars in panels A and B denote standard errors of the means. (**c**) Velocity thresholds for fixation offset versus fixation onset. Each point represents the data of one coder. The velocity thresholds for D.N., T.C., R.H., and I.H. are much higher than those for the other coders

Another way to characterize the human coders is to model their thresholds. Many fixation and saccade classifiers (Engbert, & Kliegl, 2003; Hooge & Camps, 2013; Nyström & Holmqvist, 2010; Smeets & Hooge, 2003; Van der Steen & Bruno, 1995) use the velocity threshold method. The idea is that a sample belongs to a fixation if the velocity at that sample is lower than a certain value. If we treat the human classifications as being produced by a velocity threshold model, we can determine the alleged thresholds by looking back at the velocity signal of the stimulus eye-tracking data at the fixation start and by the end. Figure 4 shows that (1) fixation onset velocity thresholds are generally higher (8 out of 12) than fixation offset velocity thresholds, and (2) except for D.N., I.H., R.H., and T.C., the thresholds are lower than 20°/s and very similar for the remaining coders. There are clear differences between the human coders here.

### Modeled parameters: Minimum saccade amplitude

**Fig. 5:**
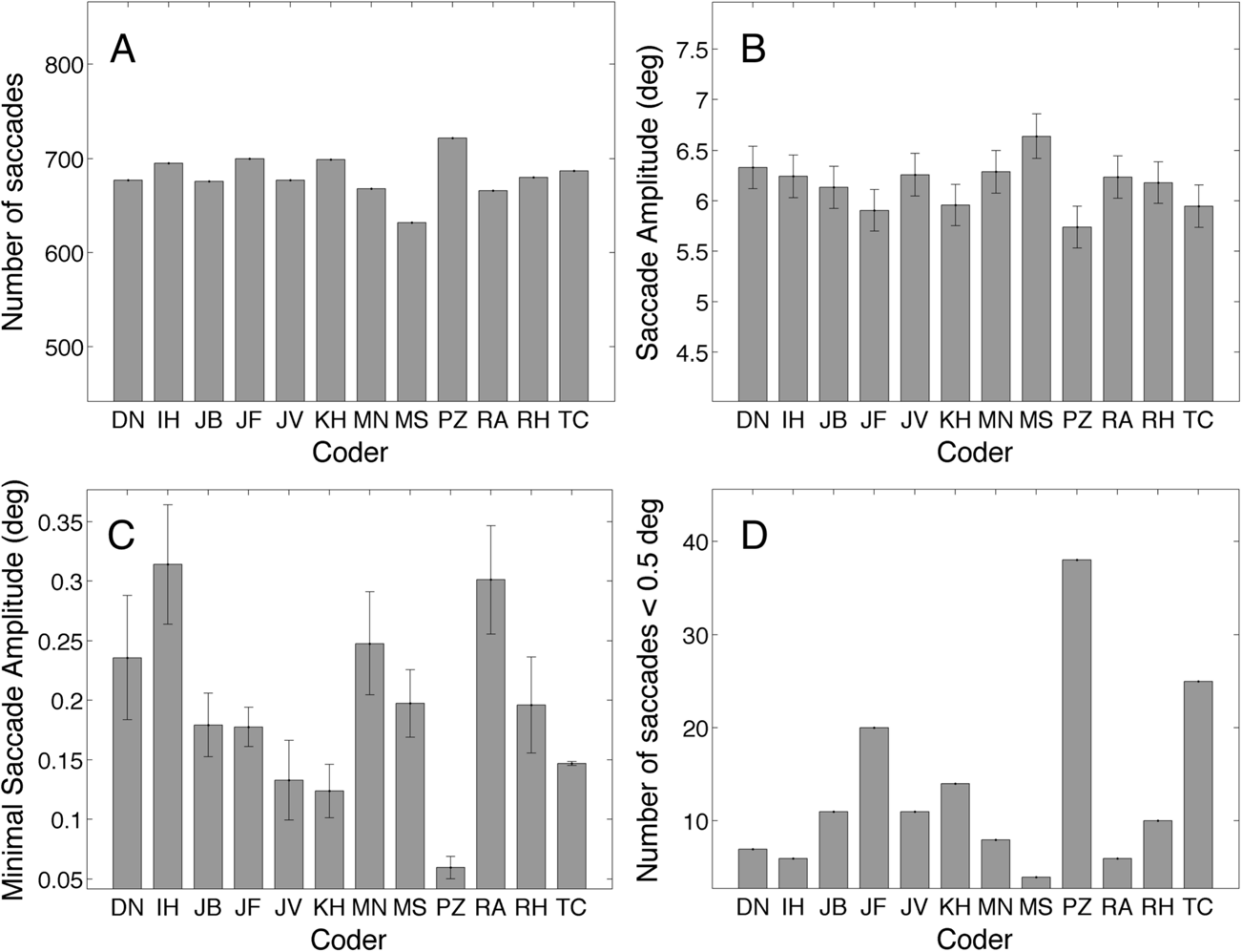
(**a**) Numbers of saccades. (**b**) Saccade amplitudes. (**c**) Mean amplitudes of the smallest five saccades and (**d**) numbers of saccades smaller than 0.5°. Error bars denote standard errors of the means

In analogy to the velocity threshold approach, we can model other thresholds. The minimal saccade threshold is another putative threshold that can be revealed from the manual classifications. We asked the coders to mark fixations and not saccades, however between the majority of the fixations, saccade candidates are located. To find these saccade candidates we took periods of data between fixations with durations shorter than 100 ms and no data loss. This duration criterion is a liberal one; large 30° saccades last about 100 ms (Collewijn, Erkelens, & Steinman, 1988). From here on, we will refer to these intervals as *saccade* instead of *saccade candidate*. Figure 5 shows the characteristics of the saccades. The mean amplitude ranges from 5.7° (P.Z.) to 6.6° (M.S.), and the number of saccades ranges from 632 (M.S.) to 722 (P.Z.). Unsurprisingly, P.Z., who classified the highest number of fixations (Fig. 3b), also classified the highest number of saccades. At the same time, P.Z. has the lowest mean saccade amplitude. Coder M.S. is the opposite. M.S. has the lowest number of saccades and the largest saccade amplitude. It seems that the fixation coding of P.Z. can be characterized by a lower saccade amplitude threshold than the other coders. We estimated the saccade size threshold by taking the mean of the five smallest saccades (Fig. 4c). The minimal saccade amplitude of P.Z. is much lower that the minimal saccade amplitudes of the other coders. P.Z. also has the largest number of saccades below 0.5° (Fig. 4d).

### Dealing with data loss

The eye-tracker signal may contain empty samples (also referred to as data loss). The nature of data loss is explained as follows by Hessels, Andersson, Hooge, Nyström, and Kemner (2015a): “This might be because a participant is looking outside the tracking area (e.g., away from the screen) or because a participant’s eyelids are closed due to a blink. Data loss can, however, also occur when the participant is directed toward the screen and the participant’s eyes are open. This might be for a number of reasons: It could be that the eye tracker is unable to detect the eyes, the pupil, or the corneal reflection” (p. 605). Some automated fixation and saccade classifiers do not tolerate data loss in fixations. An example of such an algorithm is the original implementation of NH2010 (Nyström & Holmqvist, 2010). However, many of the algorithms deal with data loss in a rather implicit and indirect way. The I2MC fixation classifier (Hessels, Niehorster, et al., 2016b) merges subsequent fixations that are spatially closer than this distance. Between these fixation small saccades or short periods of data loss may be located. The result of the merging rule is that periods of data loss may become part of periods that are classified as fixation. The I2MC algorithm also has an explicit way of dealing with data loss; periods of lost data up to 30 samples (100 ms) are interpolated if they are flanked by at least by two valid samples at each side.

**Fig. 6:**
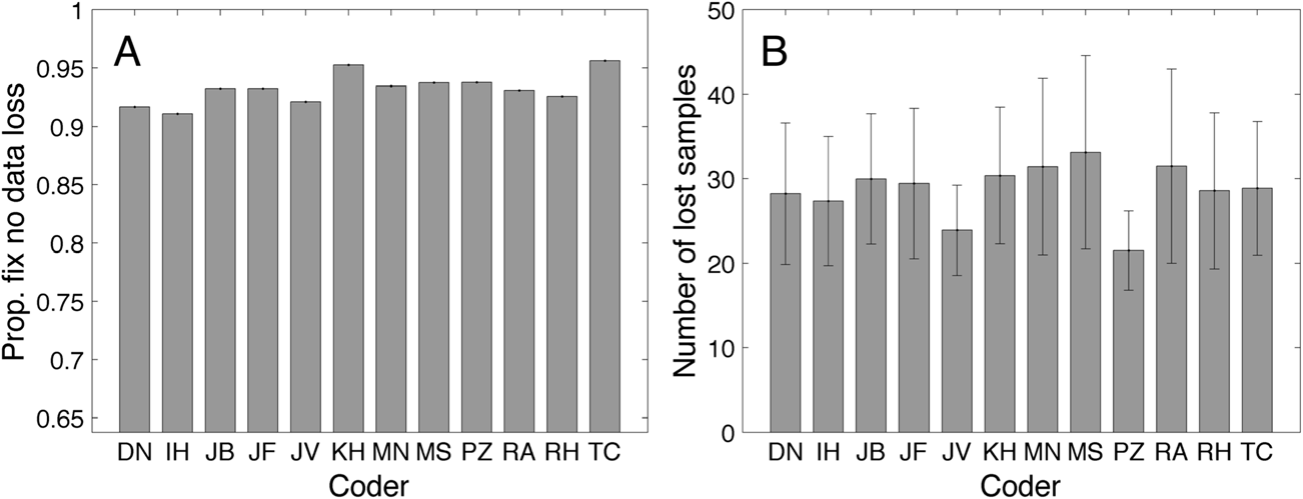
(**a**) Proportions of fixations without data loss (meaning no empty samples). (**b**) Mean numbers of lost samples per fixation in fixations with data loss. Error bars denote standard errors of the means

How do the human coders deal with data loss? Do human coders classify fixations containing data loss? Figure 6 shows the proportions of fixations without data loss; this proportion ranges from .91 (I.H.) to .96 (T.C.). All coders classify fixations with data loss, and the mean number of lost samples per fixation containing data loss ranges from 21.5 (P.Z.) to 33.1 (M.S.). Unsurprisingly, P.Z. has the lowest number of lost samples per fixation that contained data loss; he classified the highest number of fixations with the shortest fixation durations (Fig. 3). M.S. has the lowest number of fixations and the longest fixation durations, as well as the highest number of lost samples per fixation that contained data loss (Fig. 6b).

### Applying a rule to the manual classifications

**Fig. 7:**
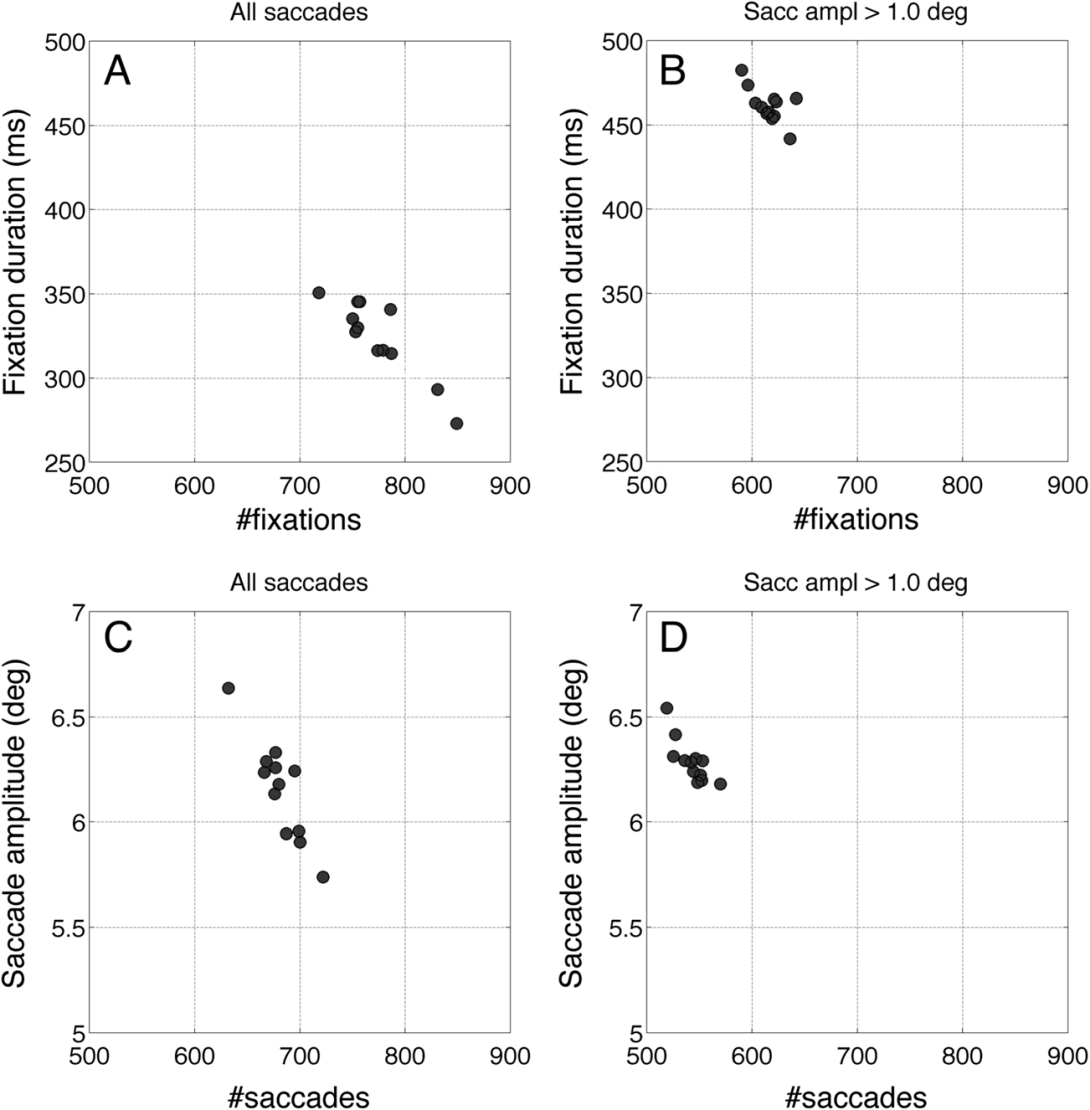
Eye movement parameters before and after removal of saccades smaller than 1°. After removal of a small saccade, the preceding and succeeding fixations were merged. Each data point represents the mean data of one coder. (**a**) Fixation duration versus number of fixations for all saccades. (**b**) Fixation duration versus number of fixations for saccades >1°. (**c**) Saccade amplitude versus number of saccades. (**d**) Saccade amplitude versus number of saccades for saccades >1°. From panels B and D, it is clear that manual classifications are more alike when small saccades are removed from the data

As can be seen in Fig. 3, the question of whether the classifications of a dozen coders agree can be answered with a simple “no.” The intercoder fixation duration differences are larger than most fixation duration differences that may occur between very different experimental conditions (Kowler, 2011; Rayner, 1998). In addition, Figs. 4 and 5 show that the different coders seem to use different (implicit) thresholds. However, if the difference in the outcome measures (fixation duration, number of fixations, etc.) is caused by the different thresholds applied, offline filtering of the classification data should be enough to eliminate these differences. To investigate this question, we removed all ends and starts of consecutive fixations enclosing saccades smaller than 1 deg. This resulted in merging fixations separated by these small saccades. Figure 7 shows the eye movement parameters (fixation duration and saccade amplitude) before and after removal of the small saccades. Fixation durations increase, the number of fixations decreases, and the outcome measures of the different coders are much closer to each other. After removal of the small saccades, the range of fixation durations of the different coders decreases from 100 to 50 ms.

### Are coders systematic over trials?

**Fig. 8:**
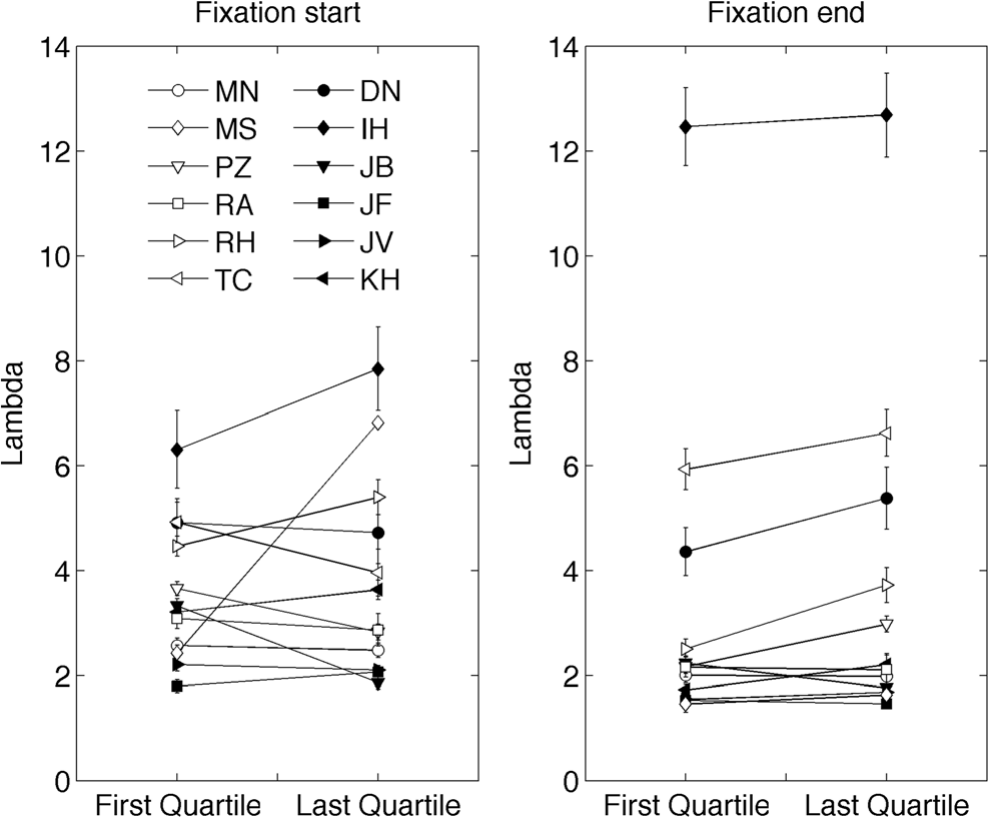
Lambda for the first and last quartiles of the fixation starts and ends. Error bars denote standard errors of the means. Error bars may be smaller than the plot symbols

Do human coders change their criteria during manual classification? We cannot simply compare the putative velocity thresholds (as in Fig. 4) from the beginning and the end of the session, because the stimuli contained varying noise levels (Table 2) and were presented in random order. Instead of the absolute velocity threshold, we use λ (Engbert & Kliegl, 2003). In the present study, λ is calculated by dividing a coders’ individual velocity threshold by the noise level of the velocity signal of the preceding fixation. In saccade detection algorithms, λ usually has a value between 2 (van der Steen & Bruno, 1995) and 6 (Engbert & Kliegl, 2003). Figure 8 shows λ for the first 25% as compared to the last 25% of the fixations classified. As is visible in the figure, λ varies a lot for the different coders, with the highest value being found for I.H. (λ = 12.7) and the lowest value for M.S. (λ = 1.5). The different coders behave differently over time. For some coders λ increases, but for others it decreases or stays constant.

### Alternative agreement measures

Cohen’s kappa captures the differences between the classifications of two coders in one number. Classifications of two coders may differ for different reasons. If we focus on the classification process, we can distinguish at least two stages. In the first stage a fixation is recognized, in the second stage the start and end of the fixation are carefully set. Based on this idea we may distinguish two different situations that may lead to a similar sample-based Cohen’s kappa for a specific pair of coders. In the first situation Cohen’s kappa is smaller than 1.0 because two coders classify similar fixations but they set the end and start points differently. In the second situation Cohen’s kappa is smaller than 1.0 because the two coders classify a different number of fixations, but the ends and starts of fixations that they both indicate, are set similar. To be able to distinguish between *classifying different events* and *setting start and endpoint of a fixation differently,* we want to have two types of measures. The first type of measure is an event-based version of the F1 score (Powers, 2015; van Rijsbergen, 1979) instead of sample-based Cohen’s kappa. We expect the F1 score to produce higher values than sample-based Cohens *kappa* because we removed the timing differences by going from a sample-based to an event-based measure. Table 4 contains the F1 scores for all combinations of the 12 coders; the F1 scores range from .88 to .97. The mean F1 scores for one coder range from .902 (P.Z.) to .951 (D.N. and M.N.). The values for the F1 score are higher than those for Cohen’s kappa but show a similar pattern. Cohen’s kappa and the F1 score are correlated (*r* = .733, *p* < .0001).

**Table 4:** Event-based F1 scores for the 12 human coders

	DN	IH	JB	JF	JV	KH	MN	MS	PZ	RA	RH	TC	Mean
DN		.96	.96	.94	.96	.95	.97	.95	.90	.96	.97	.94	.951
IH	.96		.94	.94	.94	.94	.95	.93	.90	.95	.95	.93	.939
JB	.96	.94		.94	.95	.94	.96	.93	.90	.95	.97	.94	.944
JF	.94	.94	.94		.93	.93	.94	.91	.91	.93	.94	.94	.933
JV	.96	.94	.95	.93		.94	.96	.94	.91	.95	.95	.93	.941
KH	.95	.94	.94	.93	.94		.95	.92	.91	.94	.95	.94	.937
MN	.97	.95	.96	.94	.96	.95		.95	.90	.96	.97	.94	.951
MS	.95	.93	.93	.91	.94	.92	.95		.88	.93	.94	.91	.926
PZ	.90	.90	.90	.91	.91	.91	.90	.88		.90	.90	.91	.902
RA	.96	.95	.95	.93	.95	.94	.96	.93	.90		.96	.94	.943
RH	.97	.95	.97	.94	.95	.95	.97	.94	.90	.96		.94	.950
TC	.94	.93	.94	.94	.93	.94	.94	.91	.91	.94	.94		.932

The most rightward column contains the mean event-based F1 score for each coder.

he second type of measure captures whether one coder sets the start and the end of a fixation earlier or later in time than another coder. We also want to know the variability of these specific differences. We will refer to these measures as the relative timing offset (RTO), which captures the systematic relative difference between settings of two coders and the relative timing deviation (RTD), which captures the variance in the RTO. The RTO is calculated by taking the mean of all the relative time differences of the settings of two coders. RTD is calculated by taking the standard deviation of all the relative time differences of the settings of two coders. RTO and RTD are only calculated from starts and ends of fixations that have been coded by both coders of a pair.

**Fig. 9:**
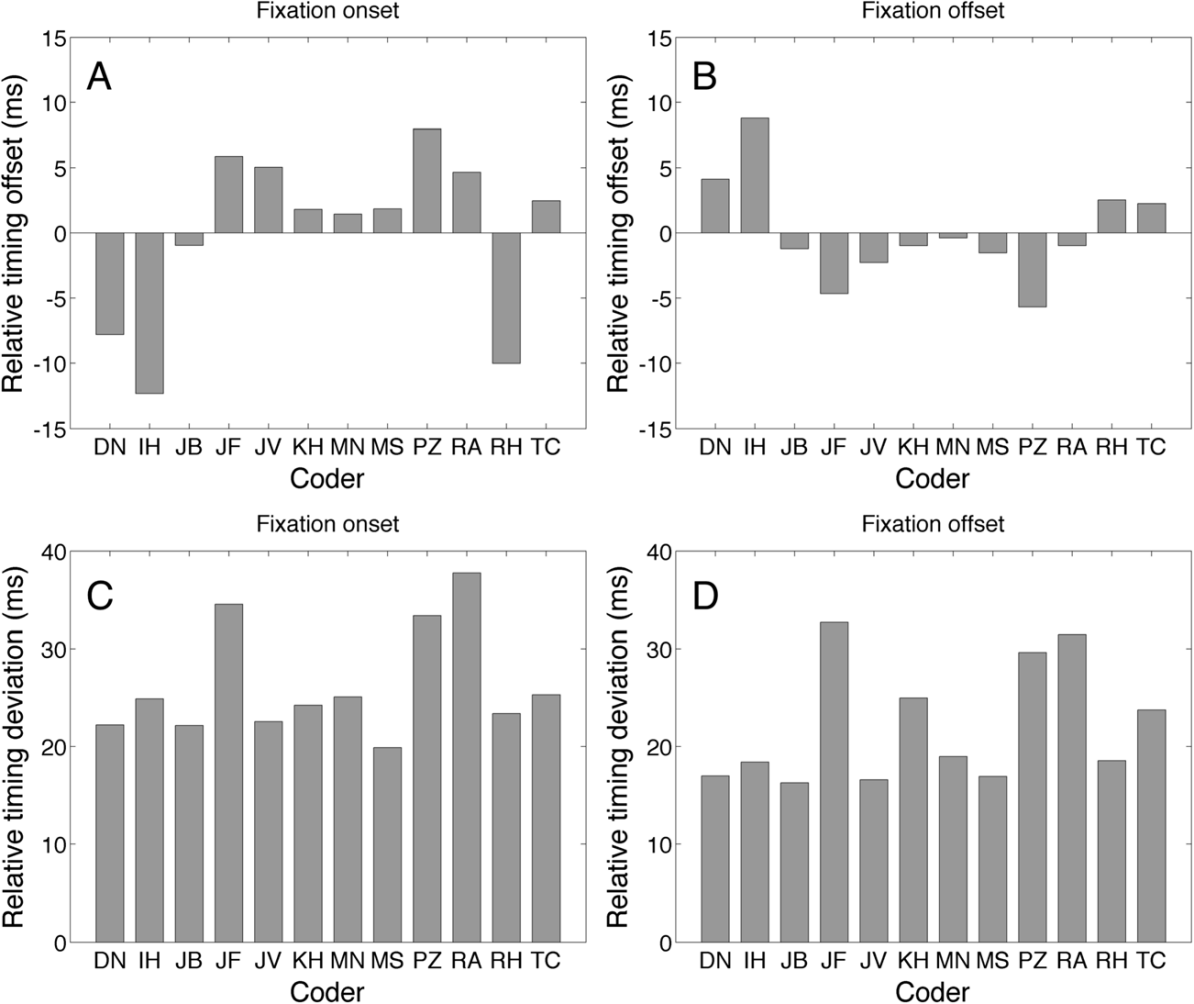
Mean relative timing offsets and mean relative timing deviations for fixation onset (**a**, **c**) and fixation offset (**b**, **d**). The means were taken over all coder combinations. A lower mean RTO indicates that this coder marked a fixation event earlier than a coder with a higher RTO. A negative mean RTO indicates that this coder marked earlier than the average coder

Figure 9 shows the mean RTO and mean RTD for fixation onset and offset. RTO and RTD were averaged over all coder combinations for a specific coder. Panels A and B show that the mean RTO of fixation onset ranges from –12.3 ms (I.H.) to 8.0 ms (P.Z.), and that the RTO for fixation offset ranges from –5.7 ms (P.Z.) to 8.8 ms (I.H.). This means that I.H. sets *fixation start* early and *fixation end* late relative to the other coders, the opposite is true for P.Z. Panels C and D show that RTD ranges from 16 to 37.8 ms. The human classifications, the eye-tracking data and the MATLAB algorithm implementations for the event-based F1 score and RTO and RTD measures are available here: 10.5281/zenodo.838313

## Discussion

### Summary of results

To investigate human classification of eye-tracking events we analyzed classifications from 12 human coders. They coded fixations in 6 min of eye-tracking data obtained from adults and infants, collected with a Tobii TX300. The time required to code the data varied enormously between coders. Some coders used up to four times the amount of time per classification as other coders (Fig. 2). Coding time and number of events classified did not correlate. We used several methods to compare the classifications between coders. We started with a sample-based version of Cohen’s kappa. The advantage of this measure is that it provides us with one number for agreement. The disadvantage is that Cohen’s kappa does not give any insight in the nature of the disagreement if there is any. We found almost perfect agreement (according to the standard of Landis & Koch, 1977) between classifications of most human coders (Table 3).

Then, we looked at measures computed from the classifications, such as fixation duration and number of fixations calculated on the basis of the classifications (Fig. 3). Contrary to the perfect agreements according to Cohen’s kappa, we found large intercoder differences for mean fixation durations (up to 75 ms) and for the number of fixations (718–849). We found a high negative correlation (*r* = –.905, *p* < .001) between fixation duration and the number of fixations, meaning that the coders followed different coding strategies. Coders with longer fixations, have a smaller number of fixations, and vice versa. In the third approach to compare the manual classifications, we modeled the coder’s putative thresholds; most velocity thresholds were below 20°/s (Fig. 4c), but four coders had much larger thresholds. We also estimated the minimal saccade amplitude thresholds, and this threshold also varied between coders (Fig. 5). As expected, coders with lower saccade amplitude thresholds produced higher numbers of fixations and shorter fixation durations.

To test whether the main difference between the classifications could have been caused by different minimal saccade amplitude thresholds, we removed the small saccades from the classifications and merged fixations around saccades smaller than 1.0. After this operation fixation durations and number of fixations were much more in agreement.

To get more insight into the differences between the classifications we introduced the event-based F1 score and developed two new measures. These are: relative timing offset (RTO) and relative timing deviation (RTD). The event-based F1 score showed that the coders agreed on the events classified and RTD and RTD showed that their detailed settings in the beginning and at end of a fixation differ.

Other analyses showed that human coders allow data loss during fixations and show that not all coders were systematic over time.

### Agenda for investigating human coding

Andersson et al. (2017) discusses several factors that may influence the performance and reliability of a human coder. These are:

1. The coding interface and resolution of the data presented.
2. Expertise.
3. Lab membership. Coders from the same lab may have similar idea’s about saccades and fixations because they use the same methods and discuss data analysis during lab meetings.
4. Labels. The present oculomotor events are ill-defined human-defined labels.
5. Different instructions may lead to different classifications
6. Noise in the data. It is unknown how human coders react to data sets with different noise levels. Andersson et al. (2017) expects human coders to be able to deal with noise better than an algorithm.
7. Differences in instrumentation (eye tracker, chinrest, etc.).

We discuss our results in the context of Andersson et al.’s list. Although we did not investigate these factors systematically and our group of 12 coders is too small to allow for comparisons within the group, we obtained enough interesting observations that may add to this discussion.

### The role of the interface in classification differences

The coding interface may play an important role in the classification of eye movement events. In the present study we opted for a simple high-resolution interface with two position panels and one velocity panel. We may expect that besides the aspects of the layout and options such as zooming in and auto-scale, the choice of units on the axes, an eye image, pupil size information, *x*–*y* display and data filtering, may play a role. Our interface contained a few of the above options and we were not primarily interested in investigating these, however, we want to discuss some aspects of our interface in relation to the classifications.

#### The velocity filter

We choose the position signal presented to be the unprocessed position signal export of the Tobii TX300. The velocity signal was constructed from the position signal by a velocity filter having a window size of 23.3 ms (seven samples, three left and right of the center). Such a filter smoothens and spreads out the velocity signal, making it possible for the human coder to detect an onset earlier in time in the velocity signal than in the position signal and detect offset later in time in the velocity signal than in the position signal. This is interesting, because I.H., D.N., and R.H. indicated that they did not use the velocity signal to set the start and end of fixations. Their putative velocity thresholds are much higher than those of the other coders (Fig. 4c). Coders who use only the position signal usually see the onset of signal changes in the position signal later in in time and the offset of changes earlier in time. This effect is also clearly visible in Fig. 9; I.H., D.N., and R.H. classify fixation onset earlier (up to three samples) and fixation offset later in time (up to 10 ms, three samples). The window size of three samples to the right and three samples to the left was not an intended and motivated choice during the design of the experiment. We would choose a smaller window if we could repeat the experiment.

#### Pixels/s or degrees/s?

The velocity signal was presented in pixels per second and autoscaled so that the highest peak in the episode used the whole vertical range of the *y*-axis of the panel. Moreover, we did not inform the coders about the viewing distance, meaning that the coders only could have guessed about the saccade size in degrees and the velocity in degrees per second. However, if we look at the putative velocity thresholds, most coders that used the velocity signal (D.N., I.H., and R.H. did not) had thresholds lower than 20°/s (only T.C. has higher thresholds). A value of 20°/s is a common threshold in velocity-based algorithms (15°/s in van der Steen & Bruno, 1995; 30°/s according to SR Research, 2007). This shows that easily interpretable units are not the necessary prerequisites for humans to do fixation classification.

#### Quick interface or detailed interface?

Manual coding is a time-consuming activity. It is worth mentioning that we did not find a speed–accuracy tradeoff (Fig. 2). We were interested in whether coders who spend more time coding also classify in a more detailed manner. This is not the case; the number of classified events did not correlate with the inter-setting interval (Fig. 2). We also did not find any evidence that the quick coders (D.N., I.H., and R.H.) produced qualitatively different classifications from the slower ones (P.Z., R.A., and T.C.). On the basis of the absence of a speed–accuracy tradeoff, we advise future human coders to classify eye-tracking data quickly. We assume that the recognition of patterns in the eye-tracking signal has more in common with the automated and fast process of visual perception than with the slower process of reasoning. The interface may play a role in the pace of coding, a quick coding interface should make use of both hands. In our interface, navigating back and forth in the data was done with the left hand (the “a” and the “d” key) and setting the fixation start and end was done with a mouse click of the right hand.

### Background of the human coders

The group of coders is too small to perform statistics on the background of the coders in relation to their classifications. However, we can describe some observations qualitatively. The years of experience in eye tracking ranged from 2 to 24 years (Table 1). According to the data, this does not seem an important factor; maybe the type of experience is more important. All the coders have experience with more than one eye tracker and all of them designed or implemented event classifiers for data analysis or for experiments with gaze contingent displays. It is also important to know that they all have experience with low frequency and higher frequency eye trackers and they processed data of low and higher quality (in terms of RMS deviation and data loss).

The coders were mainly recruited from the Lund University Humanities Lab (M.N., R.A., K.H., and D.N.) and from two groups from Experimental Psychology in Utrecht (the Attention Lab: J.F., P.Z., and M.S. and the Vision group: I.H., R.H., and J.B.). The two remaining coders (T.C. and J.V.) used to be members of the vision group. These groups do not work in isolation, the two groups from Utrecht attend scientific meetings together and members of the Vision group collaborate closely with members of the Lund University Humanities Lab.

Can we recognize the classifications based on membership of the different groups? Not really, five coders whose classifications stand out from the other seven coders are I.H. and R.H. from the vision group, D.N. from Lund, T.C. from Frankfurt, and P.Z. from the Attention Lab. P.Z. stands out because he is more meticulous than the other coders. Moreover, P.Z. is several times contrasted with M.S., as they are found to be at opposite ends of the data, which is intriguing because they work in the same room. The other four coders have negative relative timing offsets at fixation start and positive relative timing errors at fixation end. We can speculate about the origin of the offset, and it seems that they paid more attention to the position signals than to the velocity signal when they determined fixation start and end. One explanation may be that these four coders have more knowledge of the Tobii TX300 signal than the others. They all are authors on recent eye-tracker comparison studies that involved the TX300 (Hessels, Cornelissen, Kemner, & Hooge, 2015b; Niehorster, Cornelissen, Holmqvist, Hooge, & Hessels, 2017). Moreover, I.H. and R.H. reported that they mainly used the *horizontal* position signal to determine fixation end and start. They both know that the RMS deviation during fixation is remarkably lower for the horizontal than for the vertical signal of Tobii TX300 (Table 2).

To systematically investigate the background of the coder in relation to the classification results, a larger group of coders is required. When investigating this, it should be interesting to have human coders from a wide variety of backgrounds in eye movement research (e.g., reading, attention, saccade dynamics, electro-physiology and more applied topics). The suggested study could also shine a light on the problem of labels for eye movement events.

### Labels and the role of instructions and event selection rules

In the field of eye tracking there are many definitions for fixations, which may differ from “pauses over informative regions of interest” (Salvucci & Goldberg, 2000) to “Miniature eye movements that relatively stabilize the retina for a prolonged posture of the eyes over an object” (Gegenfurtner, Lehtinen, & Säljö, 2011). There is not one simple definition for a fixation; some definitions are formulated as a combination of properties (duration, frequency, amount of small movements), some are functional (e.g., to help perception) or are formulated as a recipe to detect fixations. It is to be expected that human coders have different internal representations, ideas about or definitions of fixations. However, whether this affects their classifications is an open question.

According to the F1 scores, the human coders agreed on the events (Table 4), but the relative timing offsets (RTOs) clearly showed differences in the beginnings and ends of the fixations (Fig. 9). For example, I.H. is on average 10 ms (three samples) earlier at fixation onset and 10 ms later at fixation offset than the average. In addition, if we take into account the estimated putative velocity thresholds (Fig. 4c), we can conclude that our manual coders used different (implicit) models or definitions of a fixation. However, the linear relation between the fixation duration and the number of fixations showed that the difference between classifications is not a complicated one. Coder P.Z. was mentioned frequently in the Results section because he did not allow small saccades during a fixation (Fig. 5d) and classified many short fixations (Fig. 3d); coder M.S. did the opposite (Fig. 3d).

Automated event classification usually consists of two stages: detection of event candidates and selection of the detected candidates (Hessels, Niehorster, et al., 2016b). Most algorithms consist of a sensitive detector combined with selection rules to remove details up to a level that the classifications are useful for the intended statistical analysis such as counting monocular and binocular events in microsaccade research (Gautier, Bedell, Siderov, & Waugh, 2016) or comparing dwell time of the mouth and eye regions in face perception research (Hessels, Kemner, van den Boomen, & Hooge, 2016a). We expected the human coders to have similar sensitivities and hypothesized that the event selection rules they adopt were responsible for the intercoder differences. To investigate whether the main difference between the classifications may have been caused by an (explicit or implicit) minimal saccade amplitude rule, we removed saccade candidates smaller than 1.0° from the analysis and merged fixations spatially close. Removing saccades and merging fixations made the numbers of classified fixations and fixation durations remarkably similar between coders (Fig. 7), suggesting that the main difference between the coders was the maximum size of the saccade that they tolerated during the fixation. The application of the minimal saccade amplitude rule suggests that instruction in human coding may be important if one wants to reach higher intercoder agreement. With an offline event selection rule, details can be removed (e.g., small saccades and short fixations) from the human classifications, but instruction may also work in the other direction namely to include smaller fixations and shorter saccades in the classification process. If the level of classification is detailed enough but different between coders, offline selection rules can be used to achieve agreement at the cost of loss of resolution. This will only be effective if the human coders use a high enough resolution (meaning leaving enough fine-grained elements in the classification) and do not apply coarse selection rules by themselves. In this way instruction combined with an offline selection rule may be the method to achieve better agreement between coders. Another common way of achieving better agreement between human coders is explicit instruction of a selection rule. The latter approach can be found in the instruction manual for classification of psychiatric symptoms of Wing et al. (1974), whose glossary of the definitions of symptoms is the most important part of the book.

We could have taken a completely different approach. To further test the level of agreement in settings human coders are capable of reaching, we could have set out to develop consensus guidelines, by having coders iteratively rate and review each other’s settings until a consensus coding scheme is reached. It is well established that training can improve the interrater reliability of human judgment (Buijze, Guitton, van Dijk, Ring, & the Science of Variation Group, 2012; Iwarsson & Reinholt Petersen, 2012; Lundh, Kowalski, Sundberg, & Landén, 2012; Rosen et al., 2008; Sattler, McKnight, Naney, & Mathis, 2015). Therefore, in all likelihood, such training would have enhanced the reliability of the human ratings, perhaps markedly. This would have allowed us to address the question: Is human classification, after the development of, and training on, consensus guidelines, a gold standard in fixation detection? Assuming that the consensus guidelines would be published, this would also have the additional benefit of improving the reliability of such ratings for the research community generally. Since we did not do this, we can only evaluate the scoring of experienced, but untrained raters. We were not interested in this alternative question. Although it makes sense to develop consensus guidelines for coding problems in which no automated solutions exist, in our setting automated solutions do exist and then developing such a consensus coding instruction set is not so different from programming the selection rules for a classification algorithm. Every protocol that can be specified so detailed that a computer can use it to solve the problem, should be applied by a computer instead of a human, simply because the computer outperforms the human in processing speed, capacity and consistency.

### Is human classification a gold standard in fixation detection?

We used the definition of Versi (1992): “The gold standard is not the perfect test but merely the best available test” (p. 187). Our logic is the following; if we find tests that outperform human classification, human classification is not the gold standard. The problem is defining performance because its definition may depend on the context. We will discuss manual classification in the light of three applications: (1) to process eye-tracking data, (2) to validate algorithms, and (3) to teach artificial intelligence and develop algorithms. We will start by arguing why human classification is not the gold standard of fixation detection in data processing. Then we will argue that human classification still is a gold standard test for specific eye-tracking problems and therefore a useful methodology for eye-tracking research.

- ***Manual classification to process data*** In processing eye-tracking data, manual classification is not the gold standard anymore because in this field many better automated event classifiers are available; they can be found in the software sold with eye trackers, they are freely available on the web and their principles are described in the literature. Although Komogortsev, Gobert, Jayarathna, Koh, and Gowda (2010) have previously written about manual classification that “this type of classification technique is susceptible to human error and can be open for biased interpretation with limited generalizability,” we have now provided evidence for this statement. Moreover, as Komogortsev et al. stated, “it becomes extremely tedious and time consuming to analyze large quantities of data” (p. 9).

In fields were human classification until recently dominated, automated algorithms take over quickly. New classification techniques such as identification by topological characteristics (Hein & Zangemeister, 2017) and machine learning (Zemblys et al., 2017) are promising. Other new algorithms (based on classic techniques) can deal with smooth pursuit episodes (Larsson et al., 2015) or a large variety of noise levels (Hessels, Niehorster, et al., 2016b). Mobile eye tracking is a field that is currently in transition from human classification to automated coding (e.g., Munn et al., 2008; Pfeiffer et al., 2016; SensoMotoric Instruments, 2014; Tobii Pro, 2016). However, some classification problems still require manual coding, because good automated classifiers are not available. Postsaccadic oscillation (PSO) classification is such a difficult classification problem (Hooge, Nyström, Cornelissen, & Holmqvist, 2015). However, Nyström and Holmqvist (2010), Larsson et al. (2015) and Larsson et al. (2013) proposed algorithms for PSO classification.

2. ***Manual classification to validate algorithms*** Manual classification plays a prominent role in algorithm validation. We introduced the term strict gold standard approach to emphasize that in this approach human classification is assumed to be perfect. In the present study we showed that without a good definition of a fixation and a proper set of instructions, human classifications are not perfect, they may vary over time and differ over coders. Many factors that may influence the classifications such as interface and instruction are not investigated systematically and may probably influence the classifications. However, we think of at least two important roles for human classifiers in algorithm validation. With new technical developments in eye trackers such as higher measurement frequencies and lower noise levels, researchers can see the artifacts in eye-tracking data much better than before. Examples of artifacts are PSOs. PSOs in the eye tracker signal may reflect real eyeball rotations. However, PSOs may be caused by pupil motion relative to the iris (Nyström, Hooge, & Holmqvist, 2013) or may be unrealistically enlarged due to the pupil minus CR technique (Hooge, Holmqvist, & Nyström, 2016). In the latter cases we refer to PSO’s as artifacts of a pupil based video eye tracker). What lacks is a good description of a PSO and human classifications can help to develop one. We can now scrutinize the old fixation and saccade terms more closely and realize that many issues remain to be decided on before a straight-forward automated extraction can happen. In the latter process the human eye and mind are indispensable as research tools. The second reason that human classification is still important is for finding errors produced by new algorithms. The designers of these algorithms probably perform human classification all the time during testing. In the introduction of the present study we wrote that we did not see the added value of human classification in algorithm testing. In our study (Hessels, Niehorster, et al., 2016b) we already had a ground truth because we added noise to a known signal up to a level that even the human visual system cannot detect the fixations anymore. We do see the added value now. First, we acted as human coders during testing; Second, adding examples of data with manual classification can be helpful in showing the performance of a new algorithm. In this way manual coding can be useful in algorithm design without being the gold standard of fixation detection.
3. ***Manual classification to teach artificial intelligence (AI) and to develop algorithms*** Zemblys et al. (2017) wrote: “Any already manually or algorithmically detected events can be used to train a classifier to produce similar classification of other data without the need for a user to set parameters”. It would be interesting if machine learning is used to produce automated AI classifiers that have the ability to classify eye-tracking data for which no classical algorithm exists. How can we train such an AI classifier? Data of good quality can be classified by automated algorithms and human coders should only be used to code the fuzzy, problematic parts because they are good in open-ended problems. Here human coding is still the gold standard. However, this approach and the machine-learning approach in general still involve a number of problems: (A) how to deal with a training set containing human classifications that do not agree, (B) how to deal with a training set containing inconsistent human classifications of one human coder, (C) how to test whether the training set is of good quality, (D) how to formulate the problem that is solved by the AI classifier, and (E) how to transform trained machine learning instances back to human-understandable models (given that we want understanding and not just descriptive/predictive power)? In the *microsaccade* field, researchers probably prefer another classifier than in the *reading* field. This list is not complete, but the present study provides methods to test and compare classifications among and within (human and automated) classifiers in a more detailed way than before.

### Toward improved algorithm validation: RTO and RTD

According to sample-based Cohen’s kappa human classifications are in almost perfect agreement. In contrast, measures such as fixation time and number of fixations differ greatly. If one wants more than finding out which classifier is more similar to another classifier, the sample-based Cohen’s kappa is not the ideal measure. To gain more understanding of the classification process we have split the resulting classification comparison in two parts. To compare the events classified we introduced an event-based version of the F1 score that can handle fixation classifications. To compare detailed timing settings, we developed the relative timing offset (RTO) and the relative timing deviation (RTD) measures. The advantage of the latter measures is they show that two classifiers may produce similar events, but differ in the detailed timing settings. Another possibility is that the settings are comparable but that one classifier misses events. With RTO and RTD it is possible to compare classifications in a way that is more in line with the eye-tracker measures as reported in the literature, something that eye movement researchers understand more easily.

## Conclusions

On the basis of our measurements and analysis of the literature we conclude that human classification is not the gold standard in fixation detection. Temporal offsets produced by experienced but untrained human coders do not agree and are not always systematic over time. However, human classification is still important in algorithm validation. We also see a role for human classification in the field of machine learning. Human classification can be useful in detecting features of the eye-tracker signal that are ill-defined.

To replace sample-based Cohen’s kappa we suggest the use of the event-based F1 score, the relative timing offset and the relative timing deviation measures. RTO and RTD are the missing links between agreement measures such as the F1 score and the eye movement parameters. In the present study RTO and RTD are used to investigate human classification, but they can also be used for algorithm comparisons or comparisons between automated and human classification.

## Figures and Tables

**Fig. 10:**
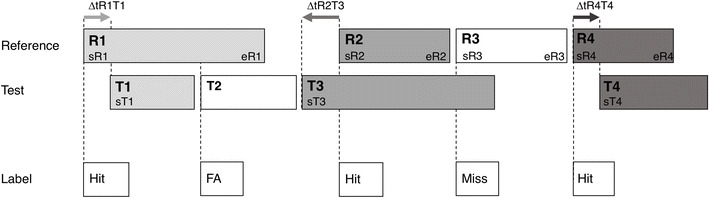
Explanation of the event-based F1 score, RTD, and RTO. Time elapses from left to right

## Appendix: Relative time offset and relative timing deviation

Here we describe how we match fixations to enable computation of the event-based F1 score, RTO and RTD. The problem at hand is drawn schematically in Fig. 10, which shows fixations of a test and a reference. To match the fixations of a reference (R1, R2, R3, . . .) and a test (T1, T2, T3, . . .), we start with reference fixation R1 (Fig. 10).

1. Find test fixations that overlap with the reference fixation. *T1 and T2 overlap with R1.*
2. The overlapping test fixation occurring earliest in time is the matching fixation for the reference fixation. The two matched fixations are labeled as hits. *R1 is matched with T1 (Fig.* *10* *), and this match is labeled as a hit*.
3. Repeat the previous for all reference fixations. *R2 is matched with T3 and R4 is matched with T4, and they are labeled as hits.*
4. Reference fixations that are not matched with test fixations are labeled as misses. *For R3 there is no match because the overlapping fixation T3 is already matched with R2. Unmatched fixation R3 is labeled as a miss.*
5. Test fixations that are not matched with reference fixations are labeled as false alarms (FA). *T2 is not matched with a reference fixation, therefore it is labeled as a false alarm.*
6. To compute the F1 score (Powers, 2011), we first count the numbers of false alarms, misses, and hits. The F1 score is calculated by the formula:

F1 = (2 * #Hits)/(2 * #Hits + #Misses + #False Alarms).

To calculate the relative timing measures (RTD and RTO) for fixation onset, we start by calculating the timing onset (Δ*t*) only for the matched fixations. For the pair of matched fixations R1 and T2 we compute Δ*t* by subtracting sR1 from sT1. The outcome (ΔtR1T1) has a positive value because T1 starts later than R1. For example, ΔtR2T3 has a negative value because T3 starts earlier than R2.

The RTD is calculated by computing the standard deviation of Δ*t*; the RTO is calculated by computing the mean Δ*t*. The recipe for computing RTO and RTD for the end of the fixation is similar to the above, except that the matching of fixations is done in the opposite order (we start at the end of the last fixation and move back in time). The RTO and RTD for fixation end are computed similarly to those for fixation start.

## Footnotes

1 We use the term *gold standard* because, according to Claassen (2005), *golden standard* is not the appropriate term.

2 CMP refers to the Comparative Manifesto Project.
